# *In vitro* screening of neuroprotective activity of Indian medicinal plant *Withania somnifera*

**DOI:** 10.1017/jns.2017.48

**Published:** 2017-10-18

**Authors:** Manjeet Singh, Charles Ramassamy

**Affiliations:** 1 INRS – Institut Armand Frappier, 531, boul. des Prairies, Laval, Québec, Canada H7V 1B7; 2 Department of Medical Biology, Faculty of Medicine, Laval University, Québec, Canada G1K 7P4

**Keywords:** *Withania somnifera*, Amyloid β-peptide, Acrolein, Oxidative stress, Acetylcholinesterase, AChE, acetylcholinesterase, AD, Alzheimer's disease, CCD, canine cognitive dysfunction, DCF-DA, 2′,7′-dichlorofluorescein diacetate, DMEM, Dulbecco's minimum essential medium, DTNB, 5,5′-dithiobis-2-nitrobenzoic acid, LDH, lactate dehydrogenase, ROS, reactive oxygen species, *WS*, *Withania somnifera*

## Abstract

Canine cognitive dysfunction (CCD) is an age-dependent neurodegenerative condition characterised by changes in decline in learning and memory patterns. The neurodegenerative features of CCD in ageing dogs and cats are similar to human ageing and Alzheimer's disease (AD). Discovering neuroprotective disease-modifying therapies against CCD and AD is a major challenge. Strong evidence supports the role of amyloid β peptide deposition and oxidative stress in the pathophysiology of CCD and AD. In both the human and canine brain, oxidative damage progressively increases with age. Dietary antioxidants from natural sources hold a great promise in halting the progression of CCD and AD. *Withania somnifera* (*WS*), an Ayurvedic tonic medicine, also known as ‘Indian ginseng’ or *ashwagandha* has a long history of use in memory-enhancing therapy but there is a dearth of studies on its neuroprotective effects. The objective of this study was to investigate whether *WS* extract can protect against Aβ peptide- and acrolein-induced toxicity. We demonstrated that treatment with *WS* extract significantly protected the human neuroblastoma cell line SK-N-SH against Aβ peptide and acrolein in various cell survival assays. Furthermore, treatment with *WS* extract significantly reduced the generation of reactive oxygen species in SK-N-SH cells. Finally, our results showed that *WS* extract is also a potent inhibitor of acetylcholinesterase activity. Thus, our initial findings indicate that *WS* extract may act as an antioxidant and cholinergic modulator and may have beneficial effects in CCD and AD therapy.

Canine cognitive dysfunction (CCD) and Alzheimer's disease (AD) are the most common age-related neurodegenerative disorders affecting millions of pets and people worldwide. It is now well established that both oxidative stress and Aβ peptide have been implicated in the pathogenesis of CCD and AD^(^^1^^)^. Aβ peptide is formed and released after the sequential cleavage of the amyloid precursor protein by β- and γ-secretases, respectively. Aβ peptide, a 40–42 amino acid-long peptide, is an important component of senile plaque formation in AD brain^(^^2^^)^ and its toxicity is mediated through the generation of hydrogen peroxide (H_2_O_2_)^(^^3^^,^^4^^)^. Reactive oxygen species (ROS), on the other hand, generate highly electrophilic α,β-unsaturated carbonyl derivatives including acrolein, 4-hydroxy-2-nonenal, and 4-oxononenal from the peroxidation of membrane lipids^(^^5^^)^. In AD brain, levels of acrolein were found to be significantly higher in several brain regions such as in the hippocampus, amygdala, middle temporal gyrus and cerebellum^(^^6^^,^^7^^)^. In primary neuronal cultures from the hippocampus, the toxicity of acrolein was higher than 4-hydroxy-2-nonenal^(^^8^^)^. We have recently established on a neuronal cell line SK-N-SH the details of the multiprocess step of the toxicity of acrolein^(^^9^^)^. We showed that, in addition to being a byproduct of lipid peroxidation, acrolein could also potentiate oxidative damage and activate several redox-sensitive pathways.

Incidence rates of age-specific AD in rural India are at least three times lower than those of an age-matched American reference population^(^^10^^)^, which could be attributed to genetic, environmental or dietary causes. *Withania somnifera* (*WS*) is one of the most important medicinal plants used in the Indian system of traditional medicine as a nootropic agent and brain tonic to restore age-related decline in mental abilities^(^^11^^)^. The major biochemical constituents of *WS* are steroidal alkaloids and steroidal lactones saponins, together called withanolides^(^^12^^)^. Presently more than twelve alkaloids and forty withanolides have been identified and characterised from the leaves, roots and berries of *Withania* species which either exist in free form, i.e. withanone, withaferin A, withanolide A, withanolides D-M, withanolides I–III, or in glycosidic form, i.e. withanosides I–VI^(^^13^^)^. Recently *WS* extract has been shown to protect against Aβ peptide- and H_2_O_2_-induced toxicities^(^^14^^–^^16^^)^. In spite of its widespread use as a brain tonic, its neuroprotective activity against acrolein remains poorly investigated.

Since multi-factorial causes have been recognised in CCD and AD as well as in other neurodegenerative disorders, CCD and AD will require multiple drug therapy to address the varied pathological aspects. Even if the strategy of combining drugs with different therapeutic targets is workable, the new pharmacological approach is to develop a multi-functional compound or extract to target multiple sites in the brain. Thus, the purpose of the present investigation was to demonstrate if a treatment with a standardised extract of *WS* can protect the human neuroblastoma cell line SK-N-SH against Aβ peptide- and acrolein-induced toxicity, decrease ROS levels and inhibit acetylcholinesterase (AChE) activity.

## Materials and methods

### Drugs and chemicals

A standardised extract of *WS* was kindly provided by Natural Remedies Private Limited, Bangalore, India. *WS* extract was certified to contain 2·6 % withanolides including withanosides IV (0·87 %) and V (0·65 %), withaferin A (0·56 %), withanolide A (0·20 %) and B (0·06 %), and 12-deoxy withastramonolide (0·26 %) as determined by HPLC. Dulbecco's minimum essential medium (DMEM), fetal bovine serum, penicillin/streptomycin, acrolein, acetylthiocholine iodide, neostigmine bromide, 5,5′-dithiobis-2-nitrobenzoic acid (DTNB), H_2_O_2_ and TOX-2 (XTT based) were obtained from Sigma-Aldrich Inc. The cytotoxicity detection kit based on the lactate dehydrogenase (LDH) assay was from Roche Diagnostics and 2′,7′-dichlorofluorescein diacetate (DCF-DA) was from Invitrogen. β-Amyloid peptide_(25–35)_ was purchased from American Peptide.

### Aβ_25–35_ preparation

The synthetic peptide Aβ_25–35_ was dissolved in sterilised tap water and incubated at 37°C for 72 h for fibrillisation before use as previously described^(^^17^^)^. Although both soluble and fibrillar Aβ peptide can induce oxidative stress, the latter is more efficient in causing lipid peroxidation^(^^18^^)^.

### Cell culture and treatment

For neuroprotective effects of *WS*, SK-N-SH cells, a human neuroblastoma cell line from American Type Cell Culture (ATCC), were maintained in DMEM supplemented with 10 % (v/v) fetal bovine serum, 100 U/ml penicillin and 100 µg/ml streptomycin. SK-N-SH cells were plated at a density of 1·5 × 10^4^ cells/well in ninety-six-well-plates (Corning) and incubated at 37°C. After 24 h of plating, the medium was completely removed and cells were kept in DMEM with antibiotics but without serum. Cells were then treated with toxic doses of Aβ (50 µg/ml) for 48 h or with acrolein (20 µm) for 24 h.

### Cell viability assays

Cytotoxicity and cell survival were measured by LDH and XTT assays by using commercial kits as previously described^(^^19^^)^. The XTT assay measures mitochondrial dehydrogenases activity and may reflect the cellular metabolic state and serves as an indicator of cell survival. Because of the proliferative effects of the *WS* extract, cells may have higher dehydrogenases activity and the XTT test alone would not be sufficient to measure the neuroprotective effects. To further confirm the protective effects of the *WS* extract, we employed the LDH test; which measures cellular membrane integrity and is a mean of quantifying dead cells.

### Intracellular reactive oxygen species level

The antioxidant activity of *WS* was confirmed by measuring its ability to scavenge ROS in SK-N-SH cells by using the fluorescent dye DCF-DA as previously described^(^^19^^)^. DCF-DA, a cell0permeable dye, is enzymically converted to a strongly fluorescent compound DCF in the presence of cellular ROS and the fluorescent intensity is directly proportional to the intracellular ROS level.

### Acetylcholinesterase inhibition

The relative AChE- inhibitory activity of *WS* extract was determined in rat brain homogenates by a spectrophotometric method^(^^20^^,^^21^^)^. The reaction mixture containing 50 µl of DTNB (3 mm), 25 µl *WS* extract, 25 µl brain homogenate from a pool of several Sprague–Dawley rats (100 µg total protein) and 100 µl of phosphate buffer. The reaction was started with the addition of 50 µl acetylthiocholine iodide (15 mm). Enzymic hydrolysis of acetylthiocholine iodide releases thiocholine, which forms a yellow 5-thio-2-nitrobenzoate anion as a result of the reaction with DTNB. The plate was read kinetically at *λ* 412 nm for 300 s and change in optical density/min was calculated. Neostigmine bromide (6·25 nm) was taken as a positive control.

### Data analysis

All results were confirmed in four or five separate independent experiments with at least three technical replicates each time and expressed as means with their standard errors. The GraphPad InStat3 program (GraphPad Software) was used for data analysis. Statistical analysis was done by one-way ANOVA followed by Dunnett's multiple means comparison test and significance was considered when *P* was <0·05.

## Results

### *Withania somnifera* extract protects SK-N-SH cells against Aβ peptide- and acrolein-induced toxicity

Results in Tables 1 and 2 show that treatment with the *WS* extract protects SK-N-SH cells against Aβ peptide- and acrolein-induced toxicity. This protection was assessed 48 h after the addition of Aβ peptide and was significant from 1·0 and 5·0 µg/ml for the LDH and XTT assays, respectively (Table 1). Interestingly, treatment with the *WS* extract could also protect SK-N-SH cells against toxicity induced by acrolein from 12·5 µg/ml of *WS* and reached a maximum at 25 µg/ml (Table 2). To rule out any false-positive effects and interference with the XTT and LDH assays, absorbance was read and no interference was observed with the different doses of *WS* tested.

**Table 1.** Effect of treatment with *Withania somnifera* (*WS*) on SK-N-SH cell survival after 24 h with Aβ peptide (50 µg/ml) using lactate dehydrogenase (LDH) and XTT assays† (Mean values with their standard errors of four or five separate experiments performed in triplicate at least in each group)

					Aβ (50 µg/ml) + *WS* (μg/ml)					
	Control		Aβ (50 μg/ml)		WS-0·5		WS-1·0		WS-5·0	
Assay	Mean	sem	Mean	sem	Mean	sem	Mean	sem	Mean	sem
LDH	100·0**	3·61	134·32	6·32	122·11	4·10	114·25**	3·79	109·28**	2·80
XTT	100·0**	4·98	42·31	6·08	62·17	7·32	69·03	13·75	71·87*	4·03

Mean value was significantly different from that of the Aβ group: * *P* < 0·05, ** *P* < 0·01.

† Results are expressed as percentage of control (taken as 100 %).

**Table 2.** Effect of treatment with *Withania somnifera* (*WS*) on SK-N-SH cell survival after 24 h with acrolein (20·0 µm) using lactate dehydrogenase (LDH) and XTT assays† (Mean values with their standard errors of four or five separate experiments performed in triplicate at least in each group)

					Acrolein (20 µm) + *WS* (μg/ml)			
	Control		Acrolein (20 µm)		WS-12·5		WS-25·0	
Assay	Mean	sem	Mean	sem	Mean	sem	Mean	sem
LDH	100·0**	4·42	215·24	7·37	139·73**	9·90	127·21**	5·96
XTT	100·0**	2·23	48·92	9·95	80·78**	6·37	81·67**	1·05

** Mean value was significantly different from that of the acrolein group (*P* < 0·01).

† Results are expressed as percentage of control (taken as 100 %).

### *Withania somnifera* extract decreases intracellular reactive oxygen species level

Since ROS and oxidative damages are involved in the toxicity of Aβ peptide and acrolein, we investigated the antioxidant activity of *WS*. The *WS* extract was able to decrease the levels of ROS in SK-N-SH cells treated with H_2_O_2_ (500 µm), with a significant effect observed from 5·0 µg/ml (Supplementary Fig. S1).

### *Withania somnifera* extract has potent acetylcholinesterase-inhibitory activity

**Fig. 1.**
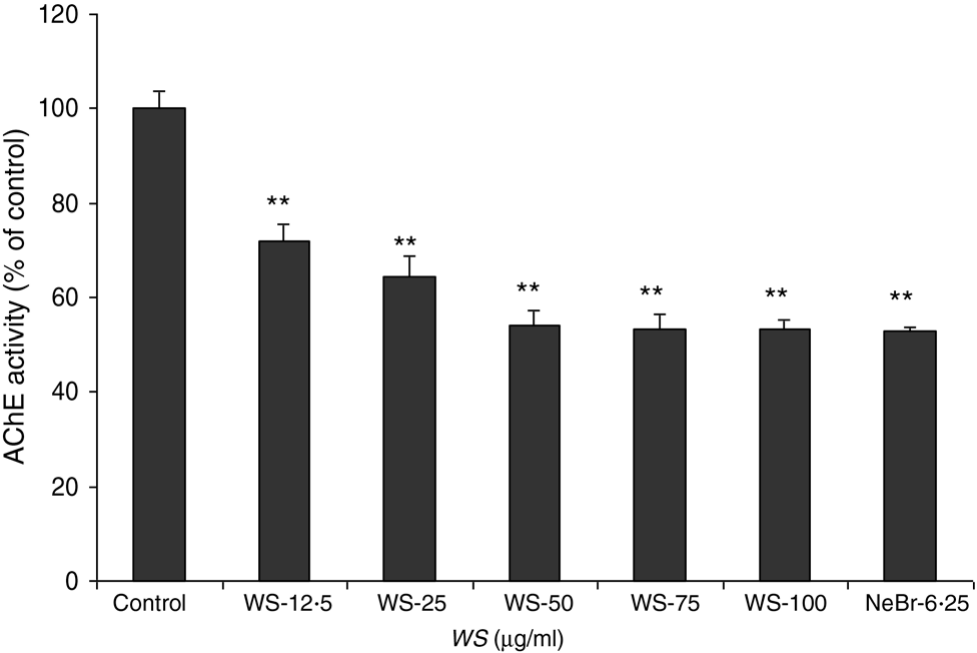
Effect of *Withania somnifera* (*WS*) extract on rat brain acetylcholinesterase (AChE) activity. Results are expressed as percentage of control (taken as 100 %). Neostigmine bromide (NeBr; 6·25 nm) was taken as a positive control. Values are means, with standard errors represented by vertical bars, of four or five separate experiments performed in triplicate at least in each group. ** Mean value was significantly different from that of the control group (*P* < 0·01).

The so-called cholinergic hypothesis of AD proposed that degeneration of cholinergic neurons in the basal forebrain and the associated loss of cholinergic neurotransmission in the cerebral cortex and other areas contribute significantly to the deterioration in cognitive function seen in patients with AD^(^^22^^)^. AChE is the key component of cholinergic synaptic transmission and plays a major role in the termination of impulse transmission by rapid hydrolysis of the neurotransmitter acetylcholine. This serves as a rationale for the use of three Food and Drug Administration-approved AChE inhibitors in the symptomatic treatment of AD^(^^23^^)^. We thus investigated the effect of *WS* on the activity of AChE on brain homogenates. As shown in Fig. 1, AChE activity was reduced by 30 % using 12·50 µg/ml of *WS* and reached about 50 % of inhibition at 50 µg/ml of *WS*.

## Discussion

There is growing interest in naturally derived bioactive compounds for pharmacological application for the treatment of CCD and AD. Different strategies are being investigated such as the neuroprotective therapeutic approach based on protection against Aβ-induced neurotoxicity and oxidative damage or inhibition of AChE. In the present study, our results showed that the *WS* extract could significantly protect SK-N-SH cells against Aβ- and acrolein-induced toxicity, decrease ROS levels in SK-N-SH cells and inhibit the activity of AChE. The protective effect against Aβ is consistent with other studies that have suggested that *WS* may be neuroprotective by mediating antioxidant effects. Similar to curcuminoids and ginkgolides, withanamides from *WS* fruit showed potent antioxidant activity as indicated by their ability to inhibit lipid oxidation^(^^24^^)^. In our study, the neuroprotective effect against Aβ could not be attributed to the effects of *WS* on ROS scavenging as its effect on ROS levels was significant from 5 µg/ml while cytoprotection was observed at lower concentrations (1 µg/ml). Other mechanisms should be investigated such as the effects of *WS* on redox-sensitive pathways because it has been demonstrated that withaferin A, the major constituents of the extract, could inhibit the activity of the transcription factors NF-κB, or AP-1^(^^25^^)^. Also, the *WS* extract was more effective than withanamide A, one of the major components of *WS*, against Aβ because withanamide A was inactive until 12·5 µg/ml^(^^15^^)^. These results indicate that other components from the *WS* extract are implicated in the neuroprotective effect against Aβ such as sominone, the active metabolite of withanoside IV^(^^14^^)^.

The present study demonstrated the protective effect of *WS* against acrolein. This result is of great interest as acrolein has been shown to be elevated in the brain from AD, from mild cognitive impairment and from preclinical AD^(^^26^^)^ and could induce neuronal and glial toxicity^(^^9^^)^. Different mechanisms could underlie this protection such as the antioxidant activity of *WS* because acrolein has been shown to increase the production of superoxide, activate NADPH oxidase activity and deplete reduced glutathione levels^(^^27^^)^. Moreover, among the α,β-unsaturated aldehydes, acrolein reacts 110–150 times faster with glutathione than 4-hydroxy-2-nonenal or crotonal^(^^9^^)^. The effects of *WS* on the activity of redox-sensitive pathways could also be involved as we have recently shown that the toxicity of acrolein modulates different signalling pathways^(^^9^^)^.

The inhibition of AChE activity by *WS* from 12·5 µg/ml described here is in line with the effects of withanolides on AChE with an half maximal inhibitory concentration (IC_50_) ranging between 20·5 and 49·2 µg/ml^(^^28^^)^.

*WS* components have been shown to induce outgrowth of axons and dendrites, and memory enhancement. Tohda & Joyashiki^(^^29^^)^ have demonstrated that sominone, an active metabolite of withanoside IV, could induce the phosphorylation of RET (rearranged during transfection), a receptor for glial cell line induced neurotropic factor (GDNF). Pharmacokinetics studies in mice suggested rapid oral absorption of withanolides and revealed that withaferin A has one and half times more relative bioavailability as compared with withanolide A^(^^30^^)^. Recent studies have shown that *WS* extract reversed brain pathology in mouse models of Alzheimer's and amyotropic lateral sclerosis, thereby indicating that certain components are bioavailable and cross the blood brain–barrier^(^^31^^,^^32^^)^.

Thus, cholinesterase-inhibiting potential along with antioxidant ability and neurotrophic and neuroprotective activity against Aβ peptide and acrolein could make *WS* extract and its constituents possible therapeutic agents in CCD, AD and senile dementia. Our investigation further strengthens the traditional use of *WS* as a nootropic agent for restoring age-related decline in mental abilities. However, further exploratory animal studies are needed to optimise therapeutic doses, to find out the right therapeutic compounds that can cross the blood–brain barrier and the duration of *WS* treatments aiming to yield desired beneficial outcomes; this further represents challenges as some of the components such as withaferin A are potentially cytotoxic^(^^33^^)^.

## Supplementary material

For supplementary material accompanying this paper visit http://dx.doi.org/10.1017/jns.2017.48. click here to view supplementary material
