# Supplementary material for: In vitro screening of neuroprotective activity of Indian medicinal plant Withania somnifera
Source: J Nutr Sci. 2017 Oct 18;6:e54. doi: 10.1017/jns.2017.48 (PMC5672322; doi:10.1017/jns.2017.48)
Supplement: Supplementary file 1 [file S2048679017000489sup001.ppt]

## Slide 1
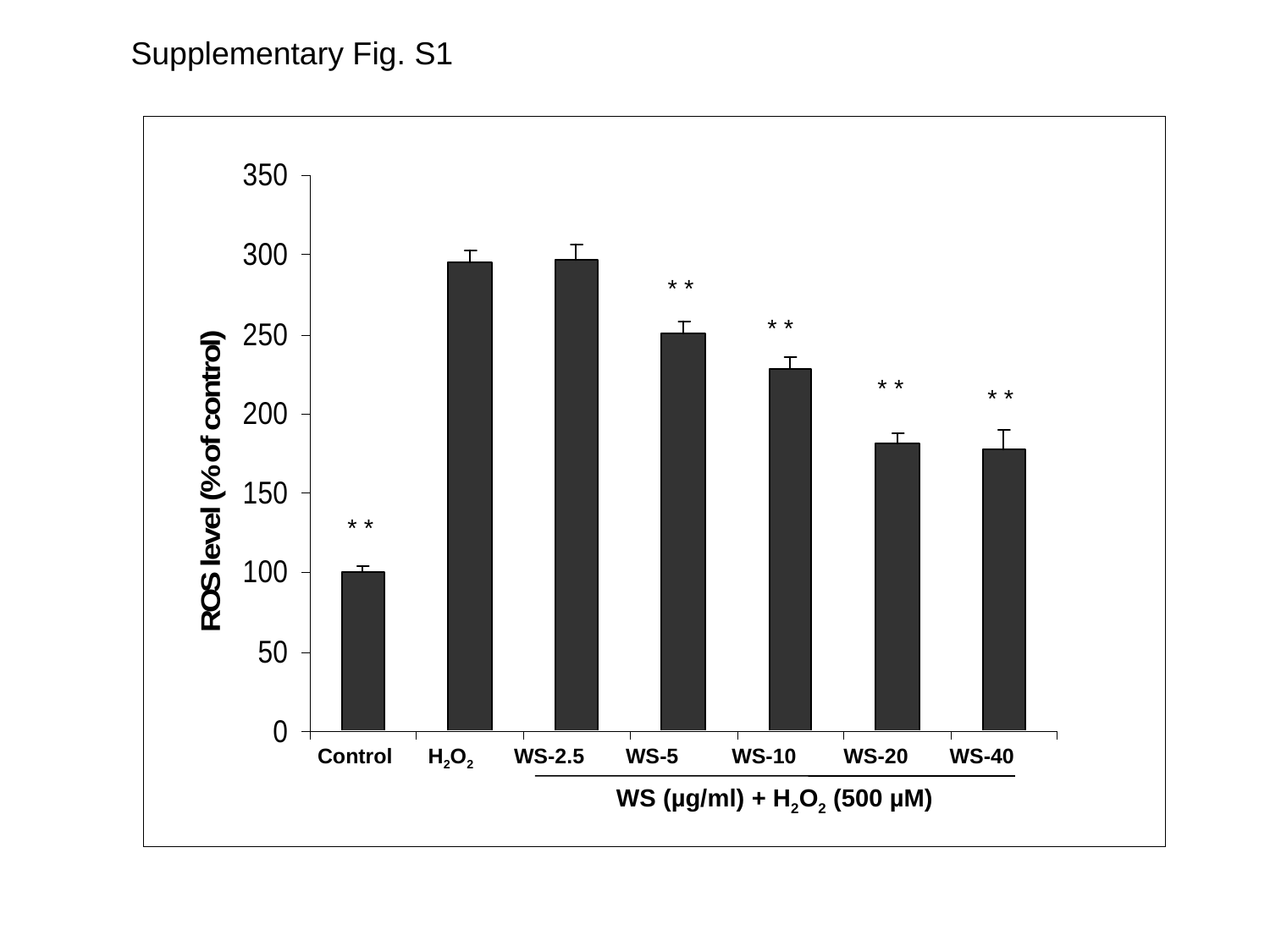

Supplementary Fig. S1
* *
* *
* *
* *
* *
Control H2O2 WS-2.5 WS-5 WS-10 WS-20 WS-40
 WS (µg/ml) + H2O2 (500 µM)

## Slide 2
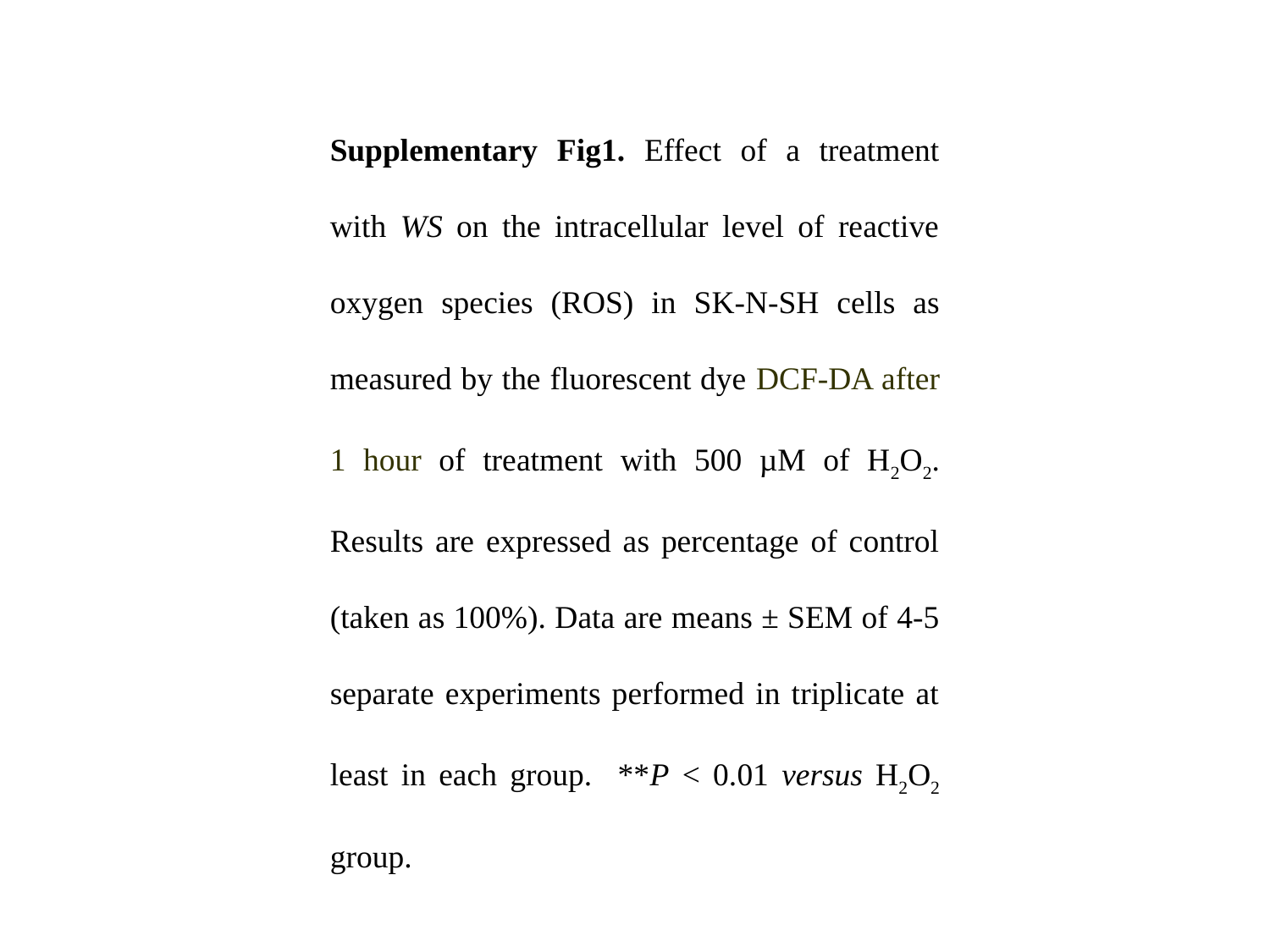

Supplementary Fig1. Effect of a treatment with WS on the intracellular level of reactive oxygen species (ROS) in SK-N-SH cells as measured by the fluorescent dye DCF-DA after 1 hour of treatment with 500 µM of H2O2. Results are expressed as percentage of control (taken as 100%). Data are means ± SEM of 4-5 separate experiments performed in triplicate at least in each group. **P < 0.01 versus H2O2 group.
